# Optimization and Benchmarking of RT-LAMP-CRISPR-Cas12a for the Detection of SARS-CoV-2 in Saliva

**DOI:** 10.3390/ijms26051806

**Published:** 2025-02-20

**Authors:** Courtney R. H. Lynch, Revel S. M. Drummond, Lauren Jelley, Lauren Baker, Erasmus Smit, Rachel Fleming, Craig Billington

**Affiliations:** 1 Institute of Environmental Science and Research Limited, Porirua 5022, New Zealanderasmus.smit@esr.cri.nz (E.S.);; 2 Plant Development, The New Zealand Institute for Plant and Food Research Limited, Auckland 1025, New Zealand; revel.drummond@plantandfood.co.nz; 3 Virology and Immunology Department, LabPLUS, Auckland City Hospital, Te Whatu Ora Te Toka Tumai, Auckland 1040, New Zealand

**Keywords:** SARS-CoV-2, RT-LAMP, CRISPR, Cas12a

## Abstract

Resource-limited settings and supply chain difficulties faced throughout the COVID-19 pandemic prompted the development of rapid and alternative methods of detecting SARS-CoV-2. These methods include reverse-transcription loop-mediated isothermal amplification (RT-LAMP), reverse-transcription recombinase polymerase amplification (RT-RPA), and CRISPR-Cas12a fluorescence detection. We describe RT-LAMP, RT-RPA, and CRISPR-Cas12a assays for the detection of the N and E-gene amplicons of SARS-CoV-2 and the optimization of various assay components, including incubation temperatures, Cas12a enzymes, reporter molecules, and the use of a lyophilized RT-LAMP master mix. We also describe the testing of a one-tube RT-LAMP-CRISPR-Cas12a assay. The one-tube assay showed promise in reducing hands-on time and improving time-to-result. We found no improvements in assay sensitivity with RT-RPA, but did achieve detection at a lower copy number with the lyophilized RT-LAMP master mix compared to liquid reagent (50 vs. 100 copies at 20 min). When used to detect the presence of SARS-CoV-2 RNA in clinical saliva samples from 75 infected patients, the discriminatory ability of the optimized RT-LAMP-CRISPR Cas12a assay was found to be comparable with RT-qPCR, with a minor reduction in sensitivity.

## 1. Introduction

Rapid point-of-use (POU) diagnostics are changing the paradigm of laboratory testing worldwide. Largely triggered by the COVID-19 pandemic, approaches which reduce time-to-result and improve access to testing are in demand. The use of isothermal amplification for the detection of SARS-CoV-2 has been increasingly adopted in place of PCR [1,2,3,4,5]. Such methods include LAMP, rolling circle amplification (RCA), and RPA. Various detection methods are available, including fluorescence, color change, or change in turbidity. To improve sensitivity and specificity, some groups have coupled isothermal amplification with CRISPR-Cas-based fluorescent detection [6,7,8,9,10].

These assays promise to deliver comparable sensitivity to qPCR, with the added advantage of results in under an hour using low-cost equipment in low-resource settings [11,12]. Although there has been a large volume of published work on rapid methods for COVID-19, few of these assays have progressed to market or clinical settings. Additionally, assays coupling isothermal amplification with CRISPR typically require separate tubes for each reaction due to their different reaction requirements. This increases sample handling and assay complexity, limiting their applicability as a POU test. Various solutions to this have been proposed, including customized tubes with separate components [7], placing one reaction in the lid of the tube [10], utilizing alternative amplification methods [13] or enzymes [14] with different conditions, avoiding the pre-amplification step by using an engineered CRISPR RNA (crRNA) to increase sensitivity [15], and microfluidic systems [2,16,17].

Here, we describe the optimization of RT-LAMP and CRISPR-Cas12a (Cas12a) assays for the detection of the N and E-gene amplicons of SARS-CoV-2 based upon the DNA Endonuclease-Targeted CRISPR Trans Reporter (DETECTR) system described by Broughton et al. (2020) [6]. The protocol described by Broughton et al. takes 30–40 min (compared to 120 min for RT-qPCR), simplifies RNA extraction, requires minimal equipment, and can be detected visually via fluorescence [6]. When compared with the ground truth (RT-qPCR), their assay was found to have 95% positive predictive agreement, and 100% negative predictive agreement. This assay contains two key components: RT-LAMP amplification of the target for 20–30 min at 62 °C, and formation of the ribonucleoprotein (RNP) complex from the specific guide RNA, Lba Cas12a, and fluorescent FAM reporter molecule (incubated for 10 min at 37 °C in a heat block). The amplified RT-LAMP product is combined with the RNP and incubated at 37 °C for 10 min, whereby trans-cleavage of the reporter molecule produces fluorescence, indicating a positive result.

Using the DETECTR approach as a baseline, we tested various method changes to determine if improvements in usability, sensitivity, and speed could be achieved. This included trialing different RT-LAMP temperatures, Cas12a enzymes (Lba and the more thermostable Yme [18]), reporter molecule sequences, amplification methods, and hardware, including an option where fluorescence is visualized by eye. The developed assay was then validated with clinical saliva samples and compared with RT-qPCR. We also describe the development of a one-tube RT-LAMP and Cas12a assay by placing the Lba Cas12a ribonucleoprotein complex in the lid of the RT-LAMP reaction tube during amplification, then shaking to mix prior to Cas12a trans-cleavage and visualization.

## 2. Results and Discussion

### 2.1. RT-LAMP Reaction Optimization

**Figure 1:**
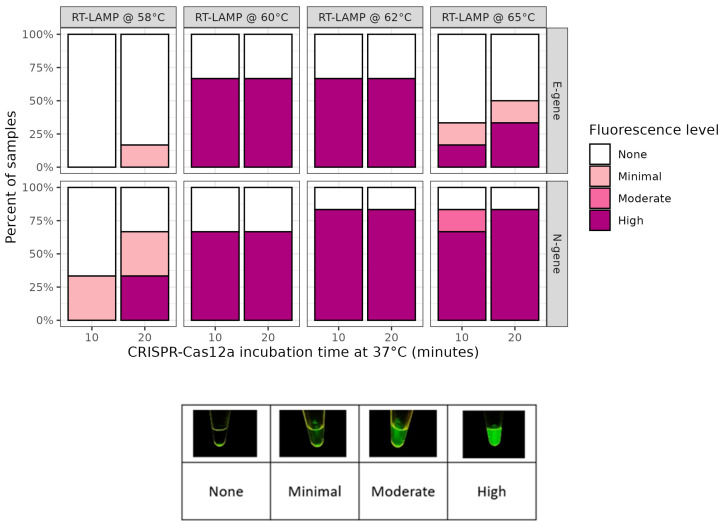
RT-LAMP optimization at various temperatures and CRISPR-Cas12a incubation times. Effect of different RT-LAMP amplification temperatures trialed with SARS-CoV-2 N- and E-gene targets and 10 or 20 min Cas12a incubation. Each bar comprises 5 omicron-positive culture material samples.

The effect of RT-LAMP incubation temperatures on the detection of SARS-CoV-2 N- and E-genes by Cas12a was tested. The level of fluorescence after 10 min of Cas12a incubation was found to vary with the RT-LAMP amplification temperature (Figure 1). No fluorescence was observed in any of the negative controls throughout this study. Minimal to no fluorescence was observed with either gene target when incubated at 58 °C. To determine if the signal could be improved at lower incubation temperatures, we doubled the Cas12a incubation time to 20 min. This improved the proportion of high fluorescence samples detected with RT-LAMP reactions at 58 °C and 65 °C but had no effect on reactions at 60 °C or 62 °C. RT-LAMP amplification at 62 °C for 20 min produced the greatest proportion of samples with high fluorescence, which shows that these were the optimal conditions among those tested (Figure 1).

### 2.2. Cas12a Reaction Optimisation

**Figure 2:**
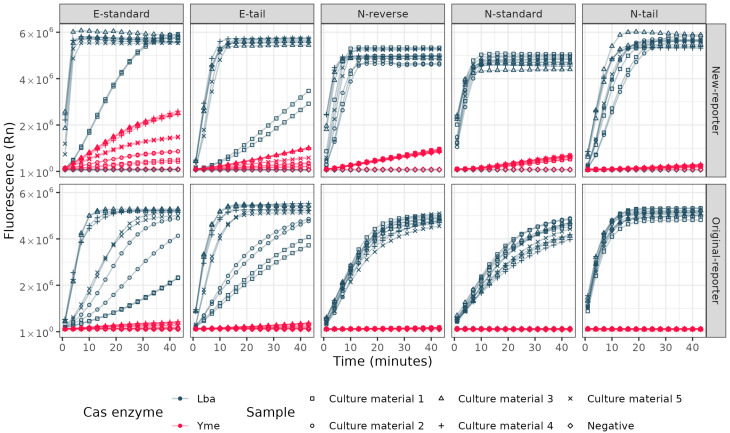
Comparison of Yme and Lba Cas12a activity with five different SARS-CoV-2 RNA guides and two different reporter molecules. Fluorescence detected via qPCR machine comparing Yme and Lba, two reporter molecules, and the guide RNA panel. RT-LAMP amplification of N- and E-gene occurred at 62 °C for 20 min. Samples were Omicron variant SARS-CoV-2 culture material. Incubation was either at 37 °C (Lba) or 55 °C (Yme) and time in min was approximated by cycle number and run time.

A key limitation when coupling RT-LAMP to Cas12a detection is that these two methods have disparate optimal incubation temperatures (60–65 °C vs. 37 °C). The use of a thermostable Cas12a would therefore be advantageous, as it may permit both reactions to be performed at the same temperature, and possibly concurrently in the same reaction. Thermostable Cas orthologs have been identified for this purpose, including Yme, Cme, Ct, SLK-9 (12a), Aap, Aac, SLK5-2 (12b) and Tcc (13a) [8,18,19,20,21]. To explore this possibility, we obtained Yme (New England Biolabs, Ipswich, MA, USA), a newly described thermostable Cas12a ortholog of the commercially available Lba Cas12a [18]. In parallel, we also explored the use of alternate RNA guide designs for SARS-CoV-2, including the addition of a 3′ tail [22,23], or a reversed binding sequence in a different location on the amplicon (this study). A real-time PCR instrument was used to quantify the comparative fluorescence levels of these combinations of Cas12a enzymes and guides (Figure 2).

Fuchs et al. reported that Yme required a higher temperature (55 °C) for trans nuclease activity and sufficient generation of fluorescent signal [18]. Even when using these conditions, we found Lba Cas12a had a much higher activity than Yme Cas12a across all targets and guides. The best results obtained for Yme were with the E-gene standard guide design, whereas for Lba, all guide RNA designs produced fluorescence, with the N-gene standard guide producing the steepest increase in fluorescence. This highlights the importance of careful guide RNA and target DNA design. Even within Lba Cas12a, variations in trans cleavage activity are observed across different targets, with a limited understanding of what features promote high activity and therefore fluorescence [24].

In addition to testing different Cas12a enzymes and guides, we also evaluated a new reporter molecule design to see if any improvements could be made on the conventional fluorophore-5′TTATT3′-quencher designs in the literature [6,9]. An in silico design approach was used, which resulted in a new reporter design (FAM-5′ATCTCGTCA-ZEN-CTCTCTCTCTCTGACGTG3′-IowaBlack; Table S1) [25]. Comparison of the fluorescence outputs from these two reporter designs was undertaken in parallel with the Yme vs. Lba comparison (Figure 2). Both Yme and Lba had higher fluorescence output levels with the new reporter molecule compared with the conventional reporter (Figure 2). Other approaches have been previously described, including dsDNA reporters, which mitigate the gradual increase in background fluorescence present with dual labeled ssDNA reporters [26]. Other reporters that adopt hairpin-like structures have subsequently been shown to also enhance activity and improve sensitivity [27].

### 2.3. Comparison of RT-RPA and RT-LAMP When Coupled to Cas12a Detection

**Figure 3:**
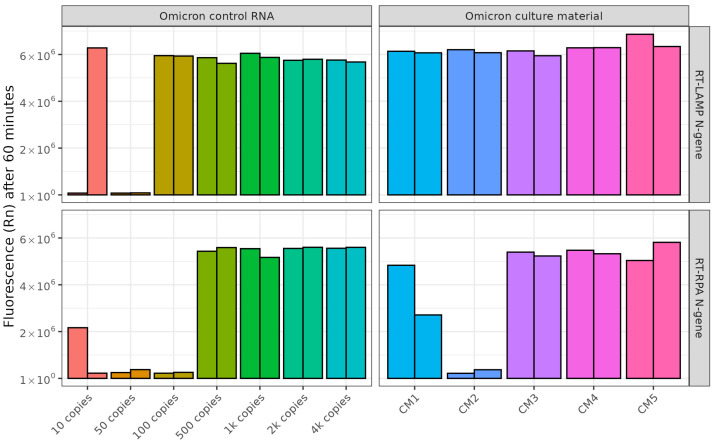
Comparison of RT-RPA and RT-LAMP coupled to Cas12a for detection of the SARS-CoV-2 N-gene. Fluorescence detected via qPCR machine with Lba (37 °C) comparing RT-RPA (40 °C for 20 min) and RT-LAMP (62 °C for 20 min) amplification of the N-gene in SARS-CoV-2 Omicron control RNA and Omicron positive culture material samples. Two replicates per sample type were tested and are shown as separate bars.

Cas12a can also be coupled to other isothermal amplification techniques such as RT-RPA [28]. The use of RT-RPA is attractive, as unlike RT-LAMP, the former can amplify targets at 37 °C which would be compatible with Cas12a in a one-tube format. We compared RT-RPA with RT-LAMP amplification of the SARS-CoV-2 N-gene using both control RNA and cell culture material, followed by Cas12a fluorescent detection (Figure 3). RT-LAMP was able to amplify a lower copy number in control samples compared with RT-RPA (100 and 200 copies per reaction, respectively). All culture material samples were successfully detected using RT-LAMP and Cas12a, whereas RT-RPA and Cas12a detected only three out of the five culture material samples (Figure 3).

A published meta-analysis of isothermal POU tests found RT-LAMP and RT-RPA have shown similar sensitivities when results are pooled [29]. There are few studies that directly compare RT-LAMP and RT-RPA for the detection of viruses. Sensitivity varied, whether this was due to primer design, target site, or amplification approach is not clear. A comparative study of Zika virus detection found RT-LAMP was able to detect 100% of samples while RT-RPA, despite possessing a faster average time to reach threshold, only detected 50% [30]. Assays to detect two viruses infecting ginger showed varied sensitivity for RT-RPA, with one assay 100× more sensitive than RT-PCR, and the other assay 1000× more sensitive, while both RT-LAMP assays were consistently 1000× more sensitive than RT-PCR [31].

### 2.4. Comparison of Liquid and Lyophilised Preparations of RT-LAMP When Coupled to Cas12a Detection

**Figure 4:**
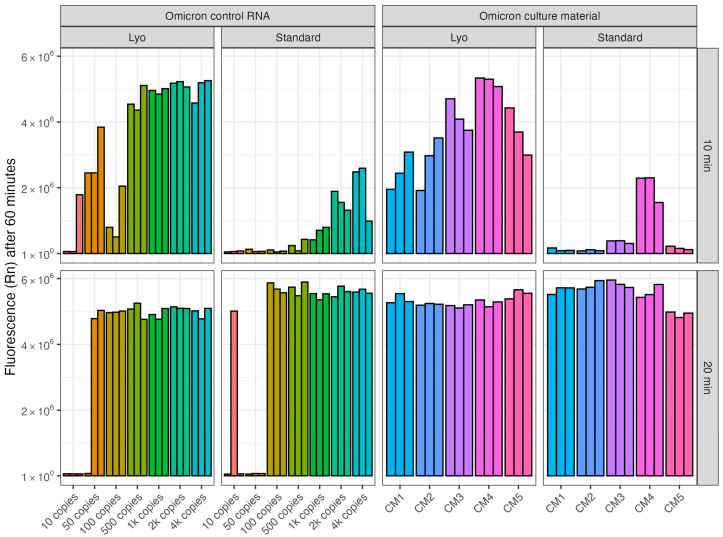
Comparison of lyophilized and standard RT-LAMP master mixes coupled to Cas12a for detection of the SARS-CoV-2 N-gene. Fluorescence detected via qPCR machine with Lba (37 °C) comparing a lyophilized (‘Lyo’) and the standard RT-LAMP master mix product (62 °C for 10 or 20 min). The N-gene was targeted in SARS-CoV-2 Omicron control RNA and Omicron positive culture material samples. Three replicates per sample type were tested and are shown as separate bars.

Lyophilized reagents offer reduced assay complexity and higher stability than liquid forms and enable storage and shipping at room temperature which is aligned with low resource field settings [32,33,34]. To test the performance of a newly developed lyophilized (Lyo) RT-LAMP master mix (NEB, Auckland, New Zealand), a comparison was made with the standard RT-LAMP master mix (NEB, Auckland, New Zealand) for amplification of the SARS-CoV-2 N-gene from both control RNA and cultured SARS-CoV-2 material, coupled with Cas12a detection. For reactions amplified for 10 min with control RNA, there was a notable difference in fluorescence detection between reagent formats (Figure 4). With the liquid RT-LAMP mix, a moderate increase in fluorescence was observed over time for some reactions (4000, 2000, and 1000 RNA copies) but for the remainder of the reactions, minimal or no fluorescence was observed. In contrast, with the Lyo mix, 500 to 4000 RNA copies were consistently detected, with some reactions positive at 10 RNA copies (Figure 4). With 20 min amplification, the detection of the N-gene was similar between the two mix formats, although somewhat improved for the Lyo mix (Figure 4). For both the Lyo and standard mixes, with 20 min amplification there was an immediate increase in fluorescence at 100 RNA copies per reaction. For the Lyo mix, some reactions at 50 RNA copies generated fluorescence, while none of the standard reaction mix gave positive results. With these assays, the use of the lyophilized mix offers greater sensitivity alongside greater alignment in a low-resource setting.

Results using SARS-CoV-2 cell culture material were comparable to those with the control RNA. With the 10 min amplification, only one of the five samples generated a low fluorescence output with the standard reagent compared to all five samples giving immediate positive results with the Lyo mix (Figure 4). With 20 min amplification, both the Lyo and standard mixes gave an immediate strong increase in fluorescence with all the SARS-CoV-2 cell culture samples (Figure 4).

Since the Lyo mix has only been released commercially recently, there are no studies published assessing its performance. However, there have been comparisons of other lyophilized nucleic acid assays for viral disease diagnostics, most of which conclude lyophilized reagents provide similar or reduced sensitivity when compared with wet reagents [33,34,35,36]. The relative advantage or disadvantage of the lyophilized reagents appear to vary depending on the type of amplification method, the assay, and the assay components [32]. However, it is important to note that some of the lyophilized reagents in these publications are a different product, rather than a lyophilized version of the wet reagents, as in this study. The Lyo mix we assessed has the added benefits of storage at room temperature, allowing for faster and more sensitive detection compared with the wet reagents, and therefore is a good fit for POU deployment [33,34].

### 2.5. RT-LAMP Target Amplification and Cas12a Detection in One-Tube

Due to their different reaction requirements (notably temperature), RT-LAMP-Cas12a assays are typically performed in a series of separate tubes. The target sequence is amplified from extracted RNA by RT-LAMP in the first tube, then the amplicons are transferred to a second tube for Cas12a-mediated detection. This two-step process increases sample handling and risk of cross-contamination and hinders useability in POU settings. We therefore tested the feasibility of RT-LAMP and Cas12a reactions in a one-tube assay. Since Yme Cas12a is reportedly active at a higher temperature (up to ~60 °C) than Lba (~37 °C) 18, we postulated that it could better survive the temperatures required for RT-LAMP (60–65 °C). In a two-tube assay, we have shown that Yme produced fluorescence when detecting the SARS-CoV-2 E-gene at both 60 °C and 62 °C, though the fluorescence intensity was lower than Lba (Figure 2). However, when tested in a one-tube format (RNP in the lid, RT-LAMP in the tube bottom; Figure S1), Yme failed to produce fluorescence. This may have been due to the premature transport of RNP from the tube lid into the RT-LAMP solution, or the heated lid of the thermocycler inactivating Yme. This was overcome by using 1.5 mL tubes in an open-top heating block, though fluorescence intensity was lower than Lba, and only observed at the maximum incubation time of 30 min (Table S2), so we decided Yme was not practical and therefore was not tested further.

**Figure 5:**
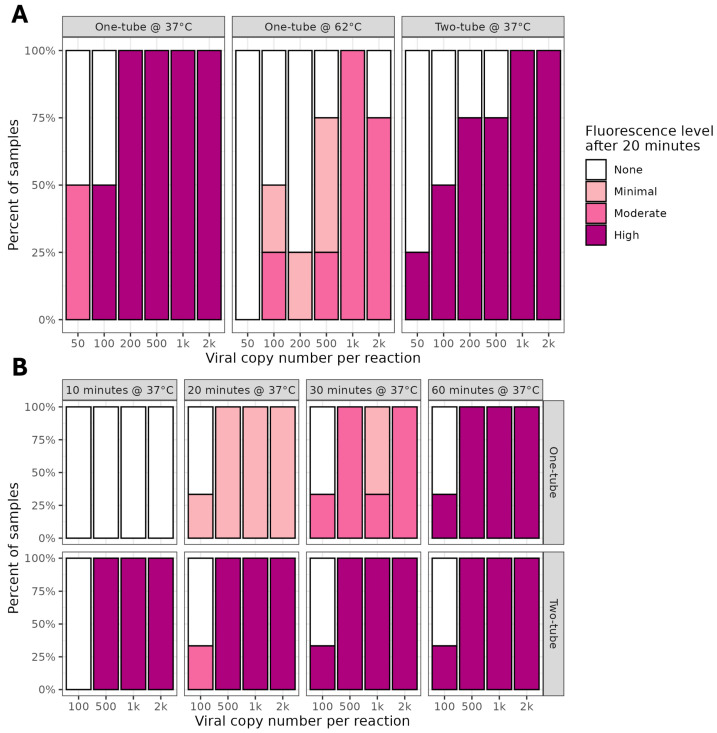
Comparison of one- and two-tube 1.5- and 0.2-mL formats for detection of SARS-CoV-2 with Lba. Proportion of samples positive and fluorescence intensities obtained in comparison of one- and two-tube E-gene assay on SARS-CoV-2 BA.2 Omicron control RNA. RNPs using Lba Cas12a and the E-gene standard guide were formed at 37 °C for 30 min and placed in the tube lid. RT-LAMP undertaken at 62 °C for 20 min. For one-tube, reaction tubes were mixed, for two-tube, RNP and RT-LAMP products were combined in a separate tube. When undertaken in 1.5 mL tubes (**A**), the header text indicates format (one- or two-tube) and final temperature for the Cas12a incubation. For 0.2 mL tubes (**B**), the header text indicates final time and temperature for the Cas12a incubation, with format (one- or two-tube) on the side panels.

The sensitivities of the one- and two-tube RT-LAMP Lba assays were compared using SARS-CoV-2 control RNA. For the one-tube assay, the final Cas12a incubation was either 37 °C or 62 °C. When incubated at 37 °C, the one-tube assay achieved comparable sensitivity to the two-tube assay, whereas at 62 °C a lower fluorescence intensity was observed, suggesting poorer sensitivity (Figure 5A, Table S3).

The use of 0.2 mL tubes had some advantages in enabling high-throughput and ease of fluorescence detection, so we revisited the one-tube reaction under these conditions. In addition, we implemented manual shaking of tubes to activate the Cas12a reaction, rather than centrifugation, to further simplify the workflow. Both assays were able to detect 500 copies of SARS-CoV-2 RNA, with the two-tube assay showing results at 10 min, compared to at least 20 min for the one-tube assay (Figure 5B, Table S4). At the lowest copy number of 100, there was inconsistent fluorescence produced by both assays and only after 60 min. Overall, the total fluorescence observed in the one-tube assays was lower in intensity than that visible in the two-tube reactions, indicating reduced sensitivity.

The temperature of the RT-LAMP reaction (62 °C) and its reagents are not optimal for the Lba Cas12a RNP and when these reactions are simply combined in the tube, no fluorescence is observed. However, we have shown that the RNP can be held in the lid of 0.2- or 1.5-mL tubes during the RT-LAMP amplification step in an open-top heat block and be activated upon mixing, but this does not work in enclosed thermocyclers. When comparing the performance of the one- and two-tube protocols, it is important to consider the total amount of product input. For the two-tube assay, 2 µL of RT-LAMP product is added to the reaction (total volume 22 µL), whereas for the one-tube protocol, the full volume of product (25 µL) is utilized in the reaction (total volume 45 µL). Despite this higher input, the sensitivity of the two methods is similar, likely due to non-optimal conditions for the RNP. In addition to the RNP being subjected to a higher temperature, the one-tube reaction contains a greater MgSO_4_ concentration (~2.5× compared with two-tube), which may not be optimal for Cas activity [18,37].

### 2.6. Comparison of Optimized RT-LAMP-Cas (Lba) Protocol with Standard RT-qPCR Assay for Detection of SARS-CoV-2 in Saliva Samples

**Figure 6:**
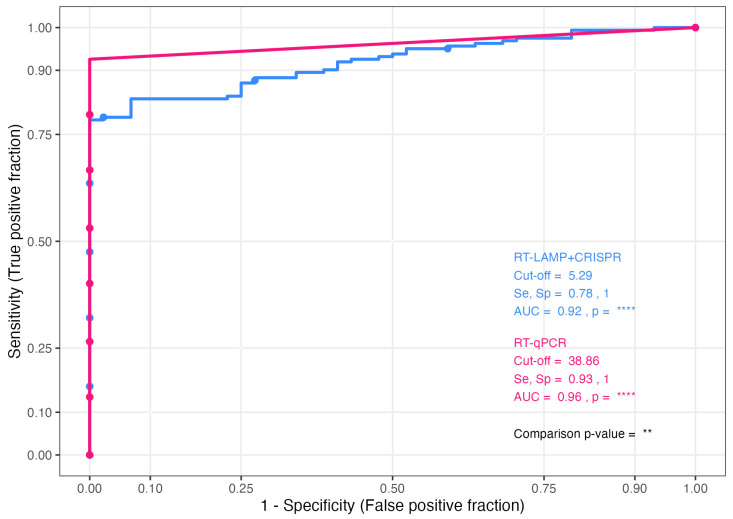
Discriminatory ability of RT-qPCR and RT-LAMP-Cas (Lba) for SARS-CoV-2 in saliva samples. Saliva samples were tested in duplicate with 5 µL input of PK lysate. For RT-qPCR, a ROC curve was created by CT values (obtained at a threshold of 0.3 normalized reporter fluorescence [ΔRn]) For RT-LAMP-Cas12 (Lba), the log10Rn (normalized fluorescence) values (obtained at endpoint/60 cycles) were used. The optimal cut-off and sensitivity and specificity at that point is shown per method. The area under the curve (AUC) is compared to an AUC of 0.5 with the results shown by the *p*-value per method. The comparison *p*-value is the result of the comparison of the AUCs for the two methods. **** *p* < 0.0001, ** *p* < 0.01.

An optimized RT-LAMP-Lba Cas12a assay, targeting the N-gene with the standard guide, was compared with the standard RT-qPCR protocol to detect SARS-CoV-2 in 75 saliva biobank samples from patients known to have COVID-19 and 21 negative controls (DEPC-treated water). Saliva samples were supplied blindly, and it was unknown whether they consisted of undiluted saliva or were in a buffer. However, there were indications that most samples were stored in buffer based on low viscosity (samples 1 to 64). Samples 65 to 75 were highly viscous and more indicative of undiluted saliva. Cas12a mediated fluorescence was detected using a qPCR machine. The results from each method were analyzed alongside the ground truth (SARS-CoV-2 positive or negative) to generate receiver operating characteristic (ROC) curves (Figure 6).

RT-LAMP-Cas (Lba) had an optimal cut-off of 5.29 log10 reduction (log10Rn) fluorescence, at which specificity was 1 and sensitivity was 0.78, meaning 78% of saliva samples from patients with COVID-19 were detected by this method (Figure 6). The area under the curve (AUC) of 0.92 indicates high discriminatory ability and was significantly different from an uninformative AUC of 0.5. RT-qPCR had an optimal cut-off of 38.86 cycles, and the specificity at this point was 1, whilst the sensitivity was 0.93. This assay showed higher discrimination between samples from COVID-19 patients and negative control samples, with the AUC of 0.96 identified as significantly different from both an AUC of 0.5 and the AUC of the RT-LAMP-Cas (Lba) assay. This observation is expected given various reports that LAMP can provide similar diagnostic performance to qPCR but is commonly less sensitive [1,38,39,40]. Bikos et al. (2024) recently demonstrated a fluorometric SARS-CoV-2 LAMP protocol which had similar sensitivity and specificity to the ‘gold standard’ RT-qPCR test in clinical samples [41]. The SHERLOCK assay, utilizing recombinase polymerase amplification coupled with Cas13 detection, has also been reported to have 100% positive agreement with RT-qPCR results in both extracted and crude SARS-CoV-2 samples [21].

A representative selection of RT-LAMP products from the biobank samples was used to examine the concordance of fluorescence data from the qPCR machine with visual interpretation of fluorescence, as may be used in POU settings. Samples were chosen to ensure a range of CT values, some positive for both methods, others positive for only RT-qPCR. In all cases, samples with log10Rn values below or close to the optimal cut-off were negative when interpreted visually (Table S5). Of interest was the comparison between saliva samples suspected to have had buffer added (samples 1–64) and those suspected to be undiluted (samples 65–75). For example, sample 12 had relatively low CT values (~25), indicating high viral copy numbers, but did not fluoresce using the RT-LAMP-Cas (Lba) method. However, sample 75 also returned similar CT values (~26), and yet the RT-LAMP-Cas (Lba) method successfully detected the N-gene, indicated by positive fluorescence. These observations corroborate findings in the literature that sample preparation and composition (e.g., undiluted versus buffered saliva) can impact RT-LAMP amplification efficiency, possibly due to incompatibility of a buffer component or pH with RT-LAMP or Cas enzyme [42]. Sample 8, where fluorescence was observed in one replicate and the CT values were around 35 cycles, is an example of the stochastic effects expected at low copy numbers.

## 3. Materials and Methods

### 3.1. Oligonucleotides and Control RNA

DNA and RNA oligonucleotides were synthesized by Integrated DNA Technologies (Coralville, IA, USA) as shown in Table S1. RT-LAMP primer and standard guide RNA sequences to amplify the N- and E-gene of SARS-CoV-2 were from Broughton et al. [6]. Vircell Amplirun^®^ SARS-CoV-2 Omicron BA.2 control RNA (Abacus dx Limited, Auckland, New Zealand) was used for assay development and sensitivity assessments. RT-qPCR primer and probe sequences were obtained from Da’an Gene Corporation (Sun Yat-sen University, Guangzhou, China).

### 3.2. SARS-CoV-2 Assay Material

Five SARS-CoV-2 Omicron lineage isolates from cell culture were obtained from the ESR Virus Identification Reference Laboratory (‘culture material samples’). All culture material samples were diluted 1:1000 prior to RT-LAMP or RT-RPA amplification. Seventy-five saliva samples from SARS-CoV-2-positive participants were obtained for testing via the ESR COVID-19 Biobank (‘Biobank samples’), with ethics approved on the 24 August 2023 by the COVID-19 Biobank Governance Group. The samples were from throughout the individual’s illness and not on a consistent day of infection. Samples were de-identified and sourced from New Zealand and overseas participants.

### 3.3. Sample Lysis

Saliva or cell culture samples (20–200 µL) were incubated with 1.8 mg/mL final concentration of proteinase K (Sigma-Aldrich, St. Louis, MO, USA) at 55 °C for 5 min to lyse viral particles, then treated at 95 °C for 5 min on a GeneAmp^®^ PCR System 9700 thermocycler (Thermo Fisher Scientific, St. Louis, MO, USA) to inactivate proteinase K. Treated samples were aliquoted and stored at −80 °C until use.

### 3.4. Ribonucleoprotein Complexes

Cas12a-guide-reporter ribonucleoprotein (RNP) complexes for each target were prepared in a master mix made for the appropriate number of samples. Per reaction: 2 µL of 10× NEB r2.1 buffer (New England Biolabs, Ipswich, MA, USA), 1 µL of 1 µM EnGen^®^ Lba Cas12a (Cpf1) or Yme Cas12a (New England Biolabs, Ipswich, MA, USA), 5 µL of 2.5 µM reporter (final concentration of 500 nM), and 1.25 µL of 1 µM guide RNA (final concentration 50 nM), were made to a final volume of 20 µL with nuclease-free water. The RNP complexes were formed at room temperature or 37 °C for 10–30 min, then stored at 4 °C for up to 24 h prior to use.

### 3.5. RT-LAMP

For RT-LAMP, a master mix was prepared per target for the appropriate number of reactions using the WarmStart^®^ Multi-Purpose LAMP/RT-LAMP 2× Master Mix (with UDG) kit (New England Biolabs, Ipswich, MA, USA). Per reaction, this included 12.5 µL of 2× NEB WarmStart Master Mix or rehydrated custom lyophilized LAMP/RT-LAMP Mix (with UDG) (New England Biolabs, Ipswich, MA, USA), 2.5 µL of 10× primer pool (final concentration F3/B3: 0.2 µM, FIP/BIP: 1.6 µM, LF/LB: 0.8 µM), and 3 µL additional 0.5 mM MgSO_4_ made to 20–23 µL with nuclease free water. Two to five µL proteinase K-treated viral RNA sample were added for a final volume of 25 µL, and the reactions incubated at 55–67 °C for 20–60 min on a thermocycler. RT-LAMP reactions were used immediately or stored at −20 °C. For experiments comparing the results with RT-RPA, reactions were scaled up to 50 µL final volume.

### 3.6. RT-RPA

A master mix was prepared to rehydrate the TwistAmp^®^ Basic (TwistDx, Ltd., Maidenhead, UK) lyophilized reactions. This included 29.5 µL Primer-Free Rehydration Buffer, 2.4 µL of Primer A (10 µM), 2.4 µL of Primer B (10 µM), 1.25 µL murine RNAse inhibitor to give 50 units per reaction (New England Biolabs, Ipswich, MA, USA), 2.5 µL RevertAid RT to give 500 units per reaction (Thermo Fisher Scientific, St. Louis, MO, USA), and nuclease free water to 45.5 µL. A two µL sample was added to the reaction mix, 2.5 µL MgOAc was added to the tube lid, and then reactions were spun down briefly for a final volume of 50 µL. This was immediately incubated for 5 min at 40 °C, inverted 10 times, spun down, and incubated for a further 15 min.

### 3.7. RT-qPCR

For RT-qPCR amplification of Biobank samples, a master mix was prepared per target for the appropriate number of reactions using the qScript XLT 1-Step RT-qPCR ToughMix kit (QuantaBio, Beverly, MA, USA). Per reaction, this included 10 µL 2× concentrated One-Step Master Mix, 1 µL Forward primer (10 µM), 2 µL Forward primer (10 µM), and 0.5 µL Probe (10 µM), made to 15 µL with nuclease free water. Five µL PK-treated viral RNA sample was added for a final volume of 20 µL in a 96-well microplate, and the reactions incubated under the following conditions using an Applied Biosystems 7500 Real-Time PCR System: 50 °C for 10 min, 95 °C for 1 min, and 45 cycles of 95 °C for 10 s, followed by 60 °C for 30 s. The cycle threshold (CT) value at a threshold of 0.3 normalized reporter fluorescence (ΔRn) was recorded.

### 3.8. Cas12a Trans-Cleavage Assay and Visualisation

For the trans-cleavage assay, 20 µL RNP and 2 µL RT-LAMP or RT-RPA product were combined and incubated at 37 °C (Lba) or 55 °C (Yme) for 10–60 min using various hardware solutions. When visualizing fluorescence using a Maestrogen blue LED illuminator (LabSupply, Dunedin, New Zealand), or the p51™ Molecular Fluorescence Viewer (miniPCR bio™, Cambridge, MA, USA), a thermoblock or thermal cycler was used. Reaction tubes were transferred to the transilluminator, and an Apple iPhone 13 mini placed on a lightbox over the viewing panel with an orange filter used to take images following incubation at various time intervals. Images were auto processed by the mobile phone application Darkroom (Apple Inc., Cupertino, CA, USA) to 0 exposure and 100 contrast to view fluorescence. When fluorescence was measured using the Applied Biosystems 7500 Real-Time PCR System (Thermo Fisher Scientific, St. Louis, MO, USA), the reactions were incubated in 96-well microplates for 45–60 cycles of: 10 s at 37 °C, and 60 s at 37 °C (data collection step), with assays labeled as FAM-NFQ MGB and no passive reference dye.

### 3.9. One-Tube Assay

For the one-tube SARS-CoV-2 assay, 20 μL of the corresponding RNP complexes were added to the lid of 0.2- or 1.5-mL tubes prior to RT-LAMP amplification, with the RT-LAMP reaction mix (prepared as described for the standard assay) in the bottom of the tube (see Figure S1). The reactions were incubated at 62 °C for 20 min on an open-top heating block. The tubes were briefly spun down and incubated at 37 or 62 °C for a further 10–30 min and imaged as described in the previous section.

### 3.10. Data Analysis

Fluorescence data collected during incubation on the Applied Biosystems™ 7500 Real-Time PCR System were exported from the HID Real-Time PCR Analysis Software v1.3. Figures were generated using R (version 4.0.3) and Posit™ Workbench (version 2021.09.0, build 351.pro6). Receiver operating characteristic (ROC) curves were generated to assess the discriminatory ability of assays using packages pROC, plotROC, and OptimalCutpoints. The area under the ROC curve (AUROC) was compared between methods using the Bootstrap method (pROC package), and to an uninformative AUROC of 0.5 using the non-parametric Mann–Whitney U-test [43,44,45].

## 4. Conclusions

We have described the successful development of an RT-LAMP and Cas12a fluorescent assay for the detection of SARS-CoV-2 in saliva samples. The optimized assay demonstrated concordance across various hardware options for the measurement of fluorescence, including the use of a transilluminator and mobile phone camera, which could be used outside of a traditional laboratory environment for point-of-use testing. Similar performance to RT-qPCR was achieved on patient saliva samples. A one-tube format was also successfully developed to simplify the assay. Future work will incorporate the lyophilized RT-LAMP master mix in the one-tube format to further improve portability and accessibility.

## Data Availability

The original contributions presented in this study are included in the article/Supplementary Materials. Further inquiries can be directed to the corresponding author.

## Supplementary Materials

The following supporting information can be downloaded at: https://www.mdpi.com/article/10.3390/ijms26051806/s1.

## Abbreviations

The following abbreviations are used in this manuscript:

MDPI	Multidisciplinary Digital Publishing Institute
POU	Point-of-use
RCA	Rolling circle amplification
RT-LAMP	Reverse-transcription loop-mediated isothermal amplification
RT-RPA	Reverse-transcription recombinase polymerase amplification
crRNA	CRISPR RNA
DETECTR	DNA Endonuclease-Targeted CRISPR Trans Reporter
Lyo	Lyophilized
RNP	Ribonucleoprotein
AUC	Area under the curve
