# Supplementary material for: Optimization and Benchmarking of RT-LAMP-CRISPR-Cas12a for the Detection of SARS-CoV-2 in Saliva
Source: Int J Mol Sci. 2025 Feb 20;26(5):1806. doi: 10.3390/ijms26051806 (PMC11899638; doi:10.3390/ijms26051806)
Supplement: Supplementary file 1 [file ijms-26-01806-s001.zip › ijms-3463435-supplementary.pdf]

Supplementary materials

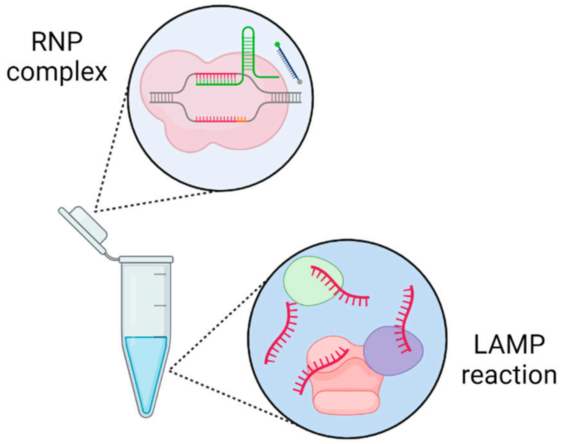

Figure S1. Reaction set-up for the one-tube RT-LAMP-CRISPR-Cas12a SARS-CoV-2 assay

Illustrated in the tube lid: RNP complex comprising Cas12a enzyme (pink), dsDNA amplicon (grey) with target sequence (red) and PAM site (orange) highlighted, RNA guide (green) and signal molecule (dark blue).

Illustrated in the bottom of the tube: reverse transcriptase (green), Bst2.0 DNA polymerase (pink/blue), primers (red).

Table S1. Primer, guide RNA and reporter sequences.

Assay name	Source	Oligo	Sequence
RT-qPCR N-gene	Da'an Gene Corporation, Sun Yat-sen University, Guangzhou, China	N-gene-forward	GGGGAACTTCTCCTGCTAGAAT
		N-gene-reverse	CAGACATTTTGCTCTCAAGCTG
		N-gene-probe	FAM-TTGCTGCTGCTTGACAGATT-TAMRA
RT-LAMP N-gene	Broughton et al. 2020	N-gene-F3	AACACAAGCTTTCGGCAG
		N-gene-B3	GAAATTTGGATCTTTGTCATCC
		N-gene-FIP	TGCGGCCAATGTTTGTAATCAGCCAAGGAAATTTTGGGGAC
		N-gene-BIP	CGCATTGGCATGGAAGTCACTTTGATGGCACCTGTGTAG
		N-gene-LF	TTCCTTGTCTGATTAGTTC
		N-gene-LB	ACCTTCGGGAACGTGGTT
		N-guide	UAAUUUCUACUAAGUGUAGAUCCCCCAGCGCUUCAGCGUUC
	This study	N-guide-reverse	UAAUUUCUACUAAGUGUAGAUCGGCCAATGTTTGTAATCAG
		N-guide-tail	UAAUUUCUACUAAGUGUAGAUCCCCCAGCGCUUCAGCGUUCUUAAUAU
RT-LAMP E-gene	Broughton et al. 2020	E-gene-F3	CCGACGACGACTACTAGC
		E-gene-B3	AGAGTAAACGTAAAAAGAAGGTT
		E-gene-FIP	ACCTGTCTCTTCCGAAACGAATTTGTAAGCACAAGCTGATG
		E-gene-BIP	CTAGCCATCCTTACTGCGCTACTCACGTTAACAATATTGCA
		E-gene-LF	TCGATTGTGTGCGTACTGC
		E-gene-LB	TGAGTACATAAGTTCGTAC
		E-guide	UAAUUUCUACUAAGUGUAGAUGUGGUAUUCUUGCUAGUUAC
	This study	E-guide-tail	UAAUUUCUACUAAGUGUAGAUGUGGUAUUCUUGCUAGUUACUUAAUAU
RT-RPA N-gene	Sun et al. 2021	N-RPA-primer-A	CAGCAGTAGGGGAACTTCTCCTGCTAGAATGG
		N-RPA-primer-B	TGGCCTTTACCAGACATTTTGCTCTCAAGCTG
		N-RPA-guide	UAAUUUCUACUAAGUGUAGAUCUGCUGCUUGACAGAUUGA
Reporter	IDT	Original reporter	/56-FAM/TT ATT/3IABkFQ/
Reporter	Drummond 2023	New reporter (R19-DQ)	/56-FAM/AT CTC GTC A/ZEN/C TCT CTC TCT CTG ACG TG/3IABkFQ/

Table S2. One-tube Yme assay in 1.5 mL tubes.

**Assay**	**Yme incubation time at 62 °C**		
	L to R: culture material 1-5, negative control		
	**10 min**	**20 min**	**30 min**
One-tube	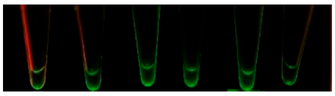	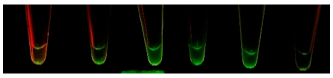	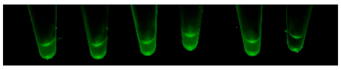
Two-tube	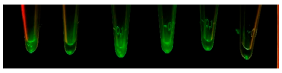	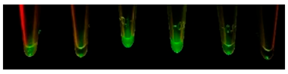	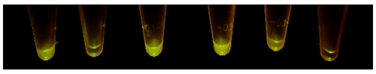

Images obtained via a transilluminator and mobile phone camera of SARS-CoV-2 Omicron culture material samples (n = 5, 1 replicate). RNP formation for 30 min at 37°C and then placed in the lid of 1.5 mL tube prior to RT-LAMP for one-tube reactions. E-gene amplified by RT-LAMP (62°C for 20 min), in one-tube reactions centrifuged to mix and in two-tube reactions combined with the RNP in a second tube. Yme incubation with E-gene standard guide at 62°C. Fluorescence recorded at three time points. Changes in colour of fluorescence are due to varied placement of the phone. Some close-up images of negative samples in the table might appear to have some fluorescence, however these are reflections which are only visible at the very top and bottom of the liquid in the tubes and are readily discriminable visually.

Table S3. Sensitivity comparison of one- and two-tube RT-LAMP-Cas (Lba) E-gene assays.

**Assay**	**Cas12a reaction 20 min**						
	Copies / reaction						
	2000	1000	500	200	100	50	0
One-tube - 62°C	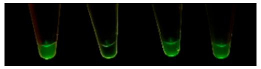	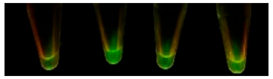	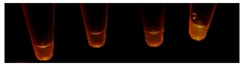	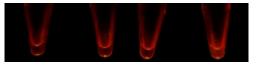	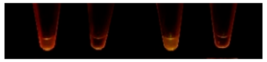	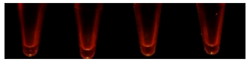	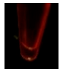
One-tube - 37°C	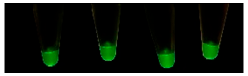	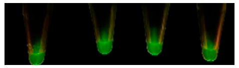	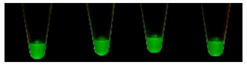	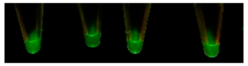	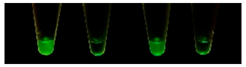	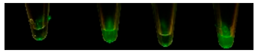	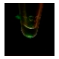
Two-tube - 37°C	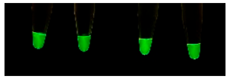	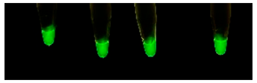	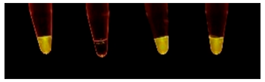	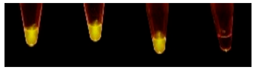	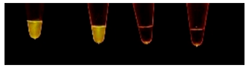	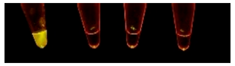	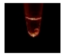

RT-LAMP at 62°C for 20 min to amplify the E-gene in a dilution series of SARS-CoV-2 BA.2 omicron control RNA with 4 replicates. The E-gene RNP formed at 37°C for 30 min and placed in the lid of the tube for one-tube reactions or combined with RT-LAMP reactions in another tube for two-tube reactions. Lba with E-gene standard guide at either 37°C or 62°C (one-tube) or solely 37°C (two-tube), images taken at 20 min. Variable colour of fluorescence is artefactual due to varied placement of the phone.

Table S4. Comparison of one- and two-tube 0.2 mL assays for detection of SARS-CoV-2 with Lba.

	Cas12a incubation at 37°C			
	**10 min**	**20 min**	**30 min**	**60 min**
**One-tube: E-gene**	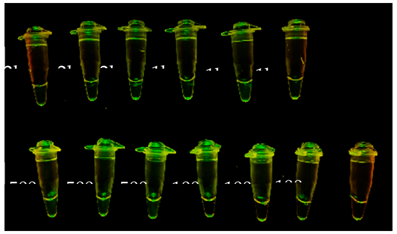	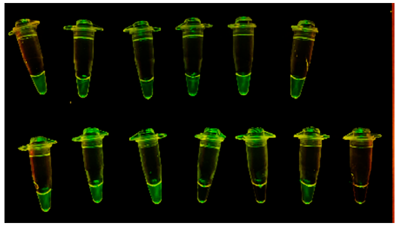	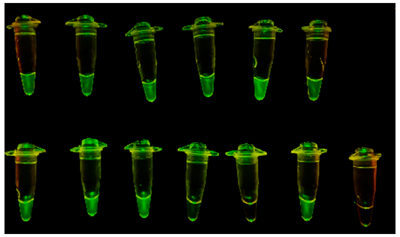	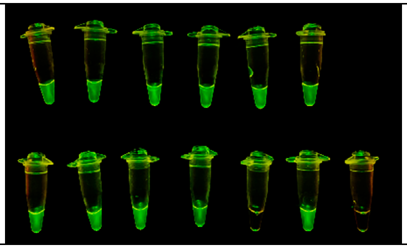
**Two-tube: E-gene**	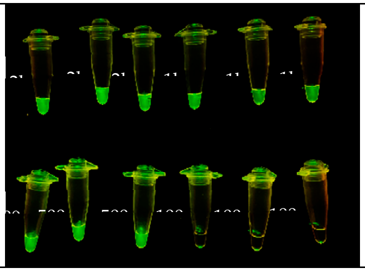	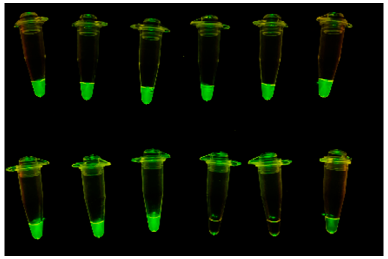	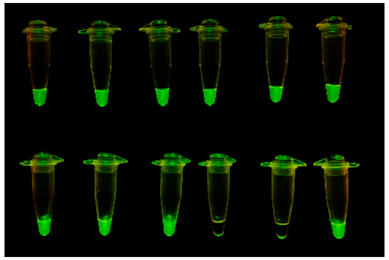	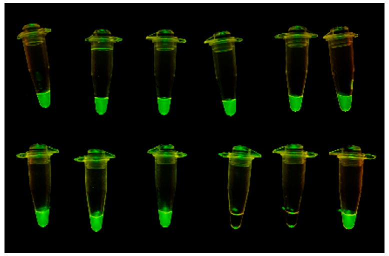

Comparison of one- and two-tube E-gene assay in 0.2 mL tubes on SARS-CoV-2 BA.2 omicron control RNA. RNP formed at 37°C for 30 min. For one tube assay, RNP placed in lid of 0.2 mL tubes prior to RT-LAMP (62°C for 20 min). One-tube reactions were shaken to mix and two-tube reactions were combined in a separate tube. Cas12a incubated at 37°C for 60 min with Lba and E-gene standard guide. Tubes are labelled with copy number per reaction.

Table S5. COVID-19 biobank RT-LAMP products visualized using transilluminator and mobile phone

Sample	Sample Fluidity	Replicate	C_T value	log_{10}Rn	Fluorescence (replicate 1: left, replicate 2: right)
8	Free-flowing	1	35.24	6.5	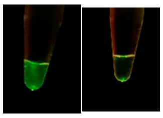
		2	35.14	6.23	
11	Free-flowing	1	31.22	4.85	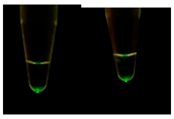
		2	30.15	5.85	
12	Free-flowing	1	25.34	5.15	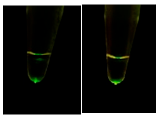
		2	24.84	4.94	
21	Free-flowing	1	18.75	6.68	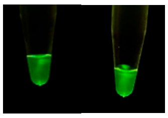
		2	18.57	6.61	
46	Free-flowing	1	23.38	6	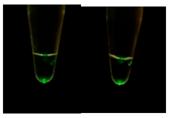
		2	23.91	5.54	
65	Viscous	1	23.16	6.65	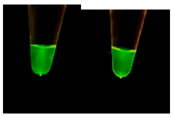
		2	22.96	6.62	
66	Viscous	1	21.29	6.65	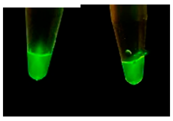
		2	21.69	6.64	
71	Viscous	1	32.36	6.62	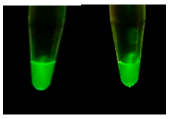
		2	32.28	6.59	
72	Viscous	1	36.92	4.77	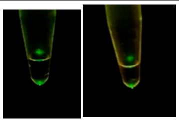
		2	n.d	5.11	
74	Viscous	1	30.38	6.61	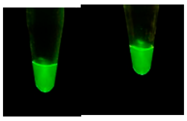
		2	31.09	6.55	
75	Viscous	1	26.9	6.65	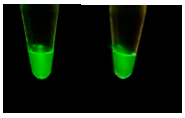
		2	26.86	6.58	
Negative control	Free-flowing	1	n.d	n.d	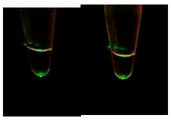
		2	n.d	n.d	

A representative selection of RT-LAMP products were visually inspected following 30 min Cas12a incubation at 37°C. The CT value was obtained using a threshold of 0.3ΔRn, and log10Rn was obtained at endpoint (60 cycles). n.d = not detected, green shading indicates a positive result was interpreted based on the image.
